# Learning temporal weights of clinical events using variable importance

**DOI:** 10.1186/s12911-016-0311-6

**Published:** 2016-07-21

**Authors:** Jing Zhao, Aron Henriksson

**Affiliations:** Department of Computer and Systems Sciences, Stockholm University, Borgarfjordsgatan 12, Kista, SE-16407 Sweden

**Keywords:** Learning weights, Temporality, Adverse drug events, Electronic health records, Machine learning, Random forest, Pharmacovigilance

## Abstract

**Background:**

Longitudinal data sources, such as electronic health records (EHRs), are very valuable for monitoring adverse drug events (ADEs). However, ADEs are heavily under-reported in EHRs. Using machine learning algorithms to automatically detect patients that should have had ADEs reported in their health records is an efficient and effective solution. One of the challenges to that end is how to take into account the temporality of clinical events, which are time stamped in EHRs, and providing these as features for machine learning algorithms to exploit. Previous research on this topic suggests that representing EHR data as a bag of temporally weighted clinical events is promising; however, the weights were in that case pre-assigned according to their time stamps, which is limited and potentially less accurate. This study therefore focuses on how to learn weights that effectively take into account the temporality and importance of clinical events for ADE detection.

**Methods:**

Variable importance obtained from the random forest learning algorithm is used for extracting temporal weights. Two strategies are proposed for applying the learned weights: weighted aggregation and weighted sampling. The first strategy aggregates the weighted clinical events from different time windows to form new features; the second strategy retains the original features but samples them by using their weights as probabilities when building each tree in the forest. The predictive performance of random forest models using the learned weights with the two strategies is compared to using pre-assigned weights. In addition, to assess the sensitivity of the weight-learning procedure, weights from different granularity levels are evaluated and compared.

**Results:**

In the weighted sampling strategy, using learned weights significantly improves the predictive performance, in comparison to using pre-assigned weights; however, there is no significant difference between them in the weighted aggregation strategy. Moreover, the granularity of the weight learning procedure has a significant impact on the former, but not on the latter.

**Conclusions:**

Learning temporal weights is significantly beneficial in terms of predictive performance with the weighted sampling strategy. Moreover, weighted aggregation generally diminishes the impact of temporal weighting of the clinical events, irrespective of whether the weights are pre-assigned or learned.

## Background

Adverse drug events (ADEs) have become a major public health problem by causing approximately 2.4 to 12.0 percent of hospital admissions worldwide [1–3]. To improve drug safety, a wide range of data sources have been used [4], among which electronic health records (EHRs) are an especially valuable source of information [5, 6]. It is not only because EHR data provides a holistic perspective of patients’ health history, including diagnoses, drug admissions, laboratory tests and so on; they also contain longitudinal data over a long time period across a large population. Nevertheless, ADEs are still under-reported in EHRs [7]. Manually screening millions of health records to identify ADEs is practically impossible for the massive amounts of data archived in an EHR database. To mitigate this problem, supervised machine learning can be adopted to automatically detect the presence of an ADE in health records in which it was not but should have been reported [8–14]. To that end, predictive models are trained to detect health records that contain ADEs with clinical events – i.e., diagnoses, drugs, clinical measurements, etc. – as features. These clinical events are reported in a chronological order in EHRs and the same event often appears in the same health record several times at different time points.

One of the advantages of using longitudinal data, such as EHRs, is that clinical events are recorded chronologically. As a result, temporality can be included as a parameter when building predictive models from this data, which is especially important for tasks like identifying ADEs. There have been attempts to handle the temporality of clinical events when fitting machine learning models to EHR data. Examples include Singh et al. [15] using EHRs to predict kidney function loss and Zhao et al. [16] identifying ADEs in EHR data. The former proposed the *Stacked-Temporal* strategy that divides patients’ medical history into a number of time windows and then merges the clinical events within each time window; this was likewise, but independently, proposed in the latter study, in which the strategy was denoted *Bag of Binned Events*. The disadvantage of this strategy is that the dimensionality of the feature increases almost proportionally with the number of time windows. Singh et al. [15] proposed an additional strategy to handle temporality, called *Multitask-Temporal*, that creates a predictive model using clinical events from each time window and aggregates the outcomes from each task. Meanwhile, Zhao et al. [16] proposed a strategy, called *Bag of Weighted Events*, that assigns weights to clinical events from different time windows and then aggregates the weighted events. Both studies use aggregation of clinical events at different time points and compare their strategy to a baseline which does not take temporality into account, and both have shown improved predictive performance when incorporating temporal information in the medical history of patients. However, in the *Multitask-Temporal* strategy, the predictive task is divided into a set of parallel tasks focusing on each time window, and, consequently, models the relationship between the target event, in their case kidney function loss, and clinical events from different time windows independently. Tackling tasks like ADE detection requires the ability to take into account all events from the medical history of patients since an ADE could be the result of a combination of clinical events at different time points. This is especially true for chronic or dose-dependent ADEs. Therefore, the *Bag of Weighted Events* strategy is advantageous in that it assigns temporal weights to clinical events from different time windows accordingly, and hence it not only takes into account the whole medical history but also incorporates the temporality of clinical events in the predictive models.

As a continuation of the *Bag of Weighted Events* strategy, another study [17] explored and evaluates various weighting strategies based on the temporality of clinical events in EHRs. Those strategies, however, obtain weights according to each clinical event’s time stamp in relation to the target event; hence each clinical event receives a weight that is calculated globally in the same way as the others, regardless of differences in terms of informativeness. For example, body temperature that is measured one day before the target event, diagnosis of hypotension, receives a higher weight compared to blood pressure from three days ago, even if the latter is considered more important in the model for predicting drug induced hypotension. Therefore, it is natural not only to weight a clinical event based on its temporality, but also, to take into account its importance in predicting the target event. This study aims to enable learning weights locally for each clinical event at each time point and evaluate their impact on the performance of predictive models for ADE detection in EHRs.

## Methods

Here, we first introduce the concept of temporal weighting from previous research; we then describe how such weights can be learned automatically. Once the temporal weights are obtained, two strategies for applying them in the predictive modeling procedure are presented. To evaluate their impact on predictive performance, a series of experiments are designed using random forest as the supervised machine learning algorithm. The experiments are conducted on 19 datasets, each one corresponding to a specific ADE, that are extracted from the Stockholm EPR Corpus, a Swedish EHR database. For each patient, a health record of 90 days before the target ADE is analyzed. To assess the predictive performance of random forest models using different temporal weighting strategies, accuracy, area under ROC curve (AUC) and area under the precision and recall curve (AUPRC) are used. Finally, the granularity of the weight learning procedure is explored in order to gain more evidence on the sensitivity of the weights learning scheme.

### Temporal weighting strategies

Temporal weighting of clinical events aims to assign a weight to each event that takes into account the temporality of this event. The assigned weight will be used to build predictive models that exploit the corresponding clinical event as a feature. In the previous study [17], a temporal weighting strategy follows such a form: for a clinical event that occurred *n* days prior to the occurrence of an ADE, weight *w* is assigned according to a curve function *f*(*n*). Nine different temporal weighting strategies were proposed in that study, all of which follow the common underlying assumption: events that occur closer to the target ADE are more important, in terms of their informativeness in the predictive models, and should hence receive more weight than those that occurred a longer time before the target ADE. Therefore, events that occurred in the same day as the target ADE receive a weight of 1, the highest weight; the weight then decreases monotonically with the number of days between the corresponding event and the target ADE until 90 days. Ninety days was chosen arbitrarily with common sense, i.e., the drugs or other clinical events that occurred more than 3 months before the occurrence of an ADE were considered unlikely to make significant contribution. All assigned weights are between 0 and 1. It was observed in that study that the impact of applying different temporal weighting strategies is significant; among the proposed strategies, the best predictive performance is obtained from assigning weights according to a reciprocal function of *n*, as follows:

1 $$  w = f(n) = \frac{1}{n}  $$

### Learning weights automatically

Learning weights automatically is, in this study, performed with the assumption that more informative clinical events should receive higher weights, in contrast to the pre-assigned weights where more recent clinical events receive higher weights. Estimation of the informativeness of clinical events with different time stamps can, naturally, be achieved by treating each clinical event from a particular time window as a separate feature, i.e., the *Bag of Binned Events* strategy in [16].

Here, we divide 90 days of the patient medical history before the target ADE event into 12 time windows, which are 1, 2, 3, 4, 5, 6, 7, 14, 21, 30, 60, 90 days before the target ADE event, excluding events from the previous time window. Therefore, the same clinical events from different time windows are treated as different features. Weights learning is then achieved by building predictive models using features from all 12 time windows and extracting weights in accordance with the informativeness of the features. In this study, this is done by calculating variable importance with the random forest algorithm [18], the variable importance is estimated using Gini score, which tests the impurity of each feature as a splitting node in the tree. The details of the random forest algorithm and variable importance is described later in *Experimental Setup*.

**Fig. 1:**
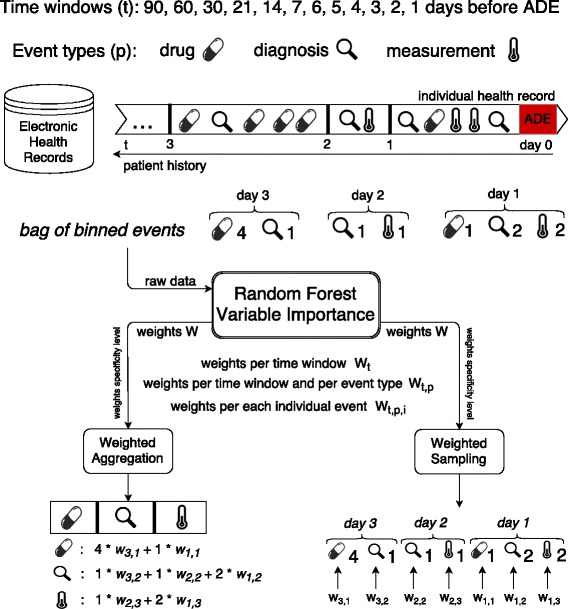
Learning temporal weights of clinical events from electronic health records
- **Weighted Sampling** – weights are used as probabilities with which features are sampled to be considered as possible splitting nodes in the random forest algorithm.

Once the weights of the clinical events have been obtained, the question moves on to how one can make use of them when building the predictive model. Here, we explore and evaluate the following two strategies for applying the learned temporal weights, which, together with the learning weights scheme, are illustrated in Fig. 1.

- **Weighted Aggregation** – corresponding weights are applied to the value of each event from different time windows and then the weighted values of the same event are aggregated.

The first strategy is a natural extension of previous studies [16, 17], while the second strategy is inspired by previous work on *enriched random forest* [19]. Although the random forest algorithm has been proven to be robust, especially in handling high dimensional data, predictive performance of the traditional random forest algorithm seems to degrade when working on high dimensional data but with a low proportion of truly informative features [19]. Such a situation typically occurs in gene expression data and longitudinal healthcare data. Here, for example, we extract all of the clinical events in the medical history of patients and use them as features describing each patient; however, it is not hard to imagine that a patient could have visited the hospital for various reasons, not all of which is related to the target ADE. In this case, among the large number of clinical events, only a small portion of them might be informative for predicting the target ADE. When the proportion of such non-informative features is large, it is almost certain that each tree in the forest would be built on at least one non-informative feature. Thus the average quality of each tree declines, and consequently results in a less robust forest. This method has been shown to be better than pre-filtering out the non-informative features on genome expression data [19]. Normally, features have an equal chance to be selected when building each tree in the forest; here, more important features have a higher chance to be selected. In this case, we use the learned weight for each feature as the probability of it being selected. Doing so allows us to avoid aggregating features from different time windows and to model their importance accordingly. It also allows us to take into account possible interactions between the same event from different time windows (see Algorithm 1).



### Granularity of weight learning

The temporal weights learned from the above scheme are specific for each individual event from each time window. To obtain insights into this scheme, in terms of sensitivity, i.e., how the predictive model’s performance would be influenced if the learned weights were from a different level of specificity, we also explored the granularity of the weight learning. More specifically, we would like to understand whether the impact of temporality varies only with the temporal information or if it is also influenced by the event types and even particular events of the same type. Here, three levels of specificity of weight learning were investigated:

- Per time window: a unique weight is learnt for all events in the same time window.
- Per event type in each time window: a unique weight is learnt for each event type (i.e., diagnosis, drug and clinical measurement) in the same time window.
- Per individual event: a unique weight is learnt for each individual event from each event type in each time window.

### Data source

In this study, 19 datasets were extracted from a real EHR system – the Stockholm EPR Corpus [20]. This research has been approved by the Regional Ethical Review Board in Stockholm (permission number 2012/834-31/5). This database contains health records from about 1.2 million patients over 7 years (2009–2015), which were collected from Karolinska University Hospital in Stockholm, Sweden. This database consists of heterogeneous types of information, including 11,623 unique diagnoses (encoded by ICD-10, the 10th revision of the International Statistical Classification of Diseases and Related Health Problems), 1,564 unique drugs (encoded by ATC, Anatomical Therapeutic Chemical Classification System), 1,877 unique clinical measurements from laboratory tests and millions of clinical notes in free-text. Here, we focus on the use of structured information, i.e., diagnoses, drugs and clinical measurements.

A previous study [21] has categorized ICD-10 diagnosis codes in terms of how they are used for indicating ADEs during hospital admissions, among which category A.1 (a drug-related causation was noted in the diagnosis code) and category A.2 (a drug- or other substance-related causation was noted in the diagnosis code) indicate a clear sign of ADE occurrence; hence the most frequent, at least assigned to 50 patients, A.1 and A.2 ADE-related diagnosis codes in the Stockholm EPR Corpus were selected. In total, 19 datasets were extracted with the existence of an ADE-related diagnosis code as the class label in each dataset. The task here is to detect patients who should, but do not, have a specific ADE reported in their health records, which results in a binary classification task.

In each dataset, positive examples are patients whom have been assigned an ADE-specific diagnosis code and negative examples are patients whom have been assigned a similar code (defined as two codes sharing the same first three levels of the ICD-10 concept hierarchy) to the corresponding ADE-related one. For instance, if the positive examples are patients diagnosed with *I95.2* (Drug-induced hypotension), the negative examples are patients diagnosed with any code starting with *I95* (Hypotension), but not *I95.2*. Features are formed from clinical events including diagnoses, drugs and clinical measurements, that occurred 90 days before the occurrence of the target ADE. According to the findings from an earlier study on representing clinical events in EHRs [8], for each example, the value for each clinical event (feature) was the *total number of times* that it occurred during the whole or a part of the patient history up to 90 days. The class label, the number of positive and negative examples and the number of clinical events involved in each dataset are described in Table 1.

**Table 1:** Datasets description including class label, number of positive examples, number of negative examples and number of related unique clinical events

ADE	Description	Positive	Negative	Events
D611	Drug-induced aplastic anaemia	593	105	2025
D642	Drug-induced secondary sideroblastic anaemia	217	9673	6076
D695	Secondary thrombocytopenia	1246	2148	4134
E273	Drug-induced adrenocortical insufficiency	70	259	1601
G620	Drug-induced polyneuropathy	96	783	2448
I952	Drug-induced hypotension	115	1287	2933
L270	Drug-induced generalized skin eruption	182	468	2480
L271	Drug-induced localized skin eruption	151	498	2481
M804	Drug-induced osteoporosis with pathological fracture	52	1170	2208
M814	Drug-induced osteoporosis	57	5097	4158
O355	Maternal care for damage to fetus by drugs	146	260	1277
R502	Drug-induced fever	80	6434	5151
T782	Adverse effects: anaphylactic shock	131	856	2639
T783	Adverse effects: angioneurotic oedema	283	720	2639
T784	Adverse effects: allergy	574	415	2635
T801	Vascular complications following infusion, transfusion and therapeutic injection	66	609	2063
T808	Other complications following infusion, transfusion and therapeutic injection	538	138	2060
T886	Drug-induced anaphylactic shock	89	1506	3765
T887	Unspecified adverse effect of drug or medicament	1047	550	3770

### Experimental setup

A series of experiments were conducted to evaluate the impact of learning weights of clinical events for predicting ADEs. First, we evaluated the predictive models using the learned temporal weights from the two proposed strategies, in comparison to using the pre-assigned weights, calculated as in Eq. 1.

In a follow-up experiment, in order to investigate how the learned weights influence the predictive performance in terms of tree quality and diversity, the average tree performance was compared to the ensemble performance, which provides some insight into the ensemble classifiers. In an ensemble model, such as random forest used in this study, there are two components that affect the predictive performance: the performance of each individual model (tree in the case of random forest) and to what extent the models vary in their predictions (often referred to as diversity [22]). In a regression framework, it is suggested that diversity can be estimated as the difference between the (squared) error of the ensemble and the average (squared) error of the ensemble members [23]. The above states that the ensemble error can be no higher than the average model error, and the more diversity, the lower the ensemble error. Currently, there is no standard for how to estimate diversity in a classification framework; here we adopt the idea from the regression framework, estimating the diversity as the difference between average tree performance and the ensemble performance in terms of error rate.

Finally, we explored the granularity of the weight learning by assigning weights on three specificity levels: (1) each individual clinical event in each time window receives a unique weight, denoted as *all*; (2) clinical events that belong to one event type in each time window receive a common weight, denoted as *type*; (3) all clinical events in each time window receive a common weight, denoted as *time*.

The random forest algorithm [18] was exploited in all experiments to generate predictive models. Random forest was chosen mainly for its reputation of being robust in terms of achieving high accuracy, its ability to handle high-dimensional data efficiently, as well as the possibility of obtaining estimates of variable importance. This algorithm is an ensemble classifier, which constructs a set of decision trees together voting for what class label to assign to an example to be classified. Each tree in the forest is built from a bootstrap replicate of the original instances, and a subset of all features is randomly sampled at each node when building the tree, in both cases to increase diversity among the trees. With increasing number of trees in the forest, the probability that a majority of trees makes an error decreases, given that the trees perform better than random and that the errors are made independently. The algorithm has often been shown in practice to result in state-of-the-art predictive performance, though this condition can only be guaranteed in theory. In this study, each random forest consisted of 500 trees. Another advantage of the random forest algorithm is that it produces variable importance, which can be estimated in different ways, see, e.g., [18]. In this study, Gini importance [24] was used as the variable importance metric, where a high Gini importance indicates that a variable plays a greater role in splitting the data into the defined classes. A Gini importance of zero means that a variable is considered useless or is never selected to build any tree in the forest.

The generated predictive models were evaluated via stratified 5-fold cross validation with 2 iterations. Performance evaluation metrics were accuracy, area under the ROC curve (AUC) and area under the precision-recall curve (AUPRC). Accuracy measures the percentage of correct predictions including both positive and negative ones. A ROC curve represents the relation between sensitivity (recall) and specificity, where the former measures how many of the positive examples have been identified as being positive and the latter measures how many of the negative examples have been identified as being negative. AUC depicts the performance of a model without regard to class distribution or error costs by estimating the probability that a model ranks a randomly chosen positive example ahead of a negative one. Both sensitivity and specificity are irrespective of the actual positive/negative balance on the test set; therefore, AUC is not biased in the case of a skewed class distribution. Finally, AUPRC represents the relation between precision and recall, depicting the probability that precision is higher than recall for each recall threshold. Since, precision measures how many of the examples been identified as being positive are true positive, and hence this score depends on how rare is the positive class. Therefore, AUPRC is preferred when the positive class is rare but of greater interest than the negative class.

When two models were compared to each other, the Wilcoxon signed-rank test was used to assess the statistical significance; when there were more than two models being compared, the Friedman test [25] was employed for statistical testing of the null hypothesis that all models perform equally, followed by a post-hoc test using the Bergmann-Hommel procedure [26] to conduct significance tests on pair-wise comparisons.

## Results

In this study, the scheme of learning temporal weights of clinical events, including two ways of applying the learned weights, i.e., *Weighted Aggregation* and *Weighted Sampling*, was evaluated. The former aggregates the same clinical event from different time windows in accordance to the learned weights, while the latter exploits the learned weights as sampling probabilities of the clinical events when constructing each tree in the random forest. In both strategies, the use of learned weights is compared to the use of pre-assigned weights for each clinical event based on its time stamp in relation to the target ADE, i.e., the number of days between the two events, respectively. Table 2 lists the results of comparing the predictive performance of the Weighted Aggregation strategy by applying pre-assigned and learned weights on 19 datasets extracted from an EHR database. It is shown that there is no significant difference on the impact of pre-assigning weights or learning weights. Nevertheless, from Table 3, where the corresponding results of the Weighted Sampling strategy are presented, we can see that by learning the temporal weights of clinical events, the predictive performance is significantly improved on all selected performance metrics. Especially for accuracy and AUPRC, performance is enhanced by about 5 % overall and for some datasets, such as D611, D695 and O355, the improvement is over 10 % on accuracy.

**Table 2:** Predictive performance of models using pre-assigned (P) or learned (L) weights in the weighted aggregation (WA) strategy

	Accuracy			AUC			AUPRC	
ADE	PWA	LWA		PWA	LWA		PWA	LWA
D611	76.46	**76.68**		0.882	0.882		**0.956**	0.954
D642	97.79	97.79		**0.973**	0.965		**0.750**	0.688
D695	76.28	**76.69**		0.863	**0.872**		**0.771**	0.768
E273	81.77	**81.78**		**0.681**	0.669		0.309	**0.323**
G620	91.64	91.64		**0.809**	0.803		0.316	**0.326**
I952	91.71	91.71		**0.583**	0.564		**0.110**	0.107
L270	**73.07**	72.12		**0.847**	0.841		**0.807**	0.793
L271	84.18	84.18		**0.774**	0.735		**0.386**	0.342
M804	95.17	95.17		**0.652**	0.615		**0.098**	0.075
M814	98.71	98.71		0.732	**0.740**		0.040	**0.050**
O355	**72.24**	71.55		0.978	**0.980**		0.934	**0.942**
R502	99.16	99.16		**0.800**	0.776		**0.218**	0.168
T782	94.81	94.81		**0.712**	0.700		**0.156**	0.152
T783	83.33	83.33		0.749	**0.758**		0.362	**0.363**
T784	66.15	66.15		0.734	**0.741**		0.809	**0.811**
T801	90.35	90.35		0.891	**0.892**		**0.493**	0.458
T808	**77.93**	77.72		**0.882**	0.879		0.958	0.958
T886	94.95	94.95		0.689	**0.692**		**0.117**	0.109
T887	**61.35**	60.97		0.751	**0.752**		**0.776**	0.773
Average	84.58	84.50		0.788	0.782		0.493	0.482
*p*-value	0.2936			0.1564			0.08743	

Bold indicates winning

**Table 3:** Predictive performance of models using pre-assigned (P) or learned (L) weights in the weighted sampling (WS) strategy

	Accuracy			AUC			AUPRC	
ADE	PWS	LWS		PWS	LWS		PWS	LWS
D611	76.46	**90.38**		0.887	**0.946**		0.949	**0.981**
D642	97.79	**99.47**		0.979	**0.983**		0.814	**0.936**
D695	71.10	**87.74**		0.895	**0.923**		0.806	**0.881**
E273	**81.78**	79.92		0.615	**0.650**		0.264	**0.272**
G620	91.64	**92.22**		0.815	**0.837**		0.334	**0.441**
I952	**91.71**	91.64		**0.557**	0.554		0.116	**0.118**
L270	67.14	**73.07**		**0.843**	0.828		0.791	**0.795**
L271	84.18	**85.22**		0.753	**0.759**		0.356	**0.406**
M804	**95.17**	95.12		0.571	**0.598**		0.072	**0.079**
M814	**98.71**	98.69		0.702	**0.722**		0.039	**0.052**
O355	82.13	**96.49**		0.980	**0.989**		0.948	**0.977**
R502	99.16	**99.22**		**0.840**	0.774		0.148	**0.280**
T782	94.81	**94.86**		0.741	**0.754**		0.188	**0.255**
T783	83.33	**86.16**		0.771	**0.788**		0.393	**0.524**
T784	66.86	**75.46**		**0.762**	0.761		**0.830**	0.829
T801	90.35	**93.55**		0.868	**0.880**		0.533	**0.635**
T808	77.93	**84.38**		0.869	**0.879**		0.954	0.954
T886	**94.95**	94.86		**0.741**	0.738		0.159	**0.190**
T887	60.19	**69.72**		0.762	**0.771**		0.765	**0.802**
Average	84.49	88.85		0.787	0.797		0.498	0.548
*p*-value	0.002838			0.01597			0.00001907	

Bold indicates winning

**Fig. 2:**
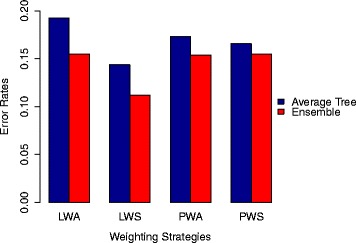
Error rates of average tree versus ensemble of the weighted aggregation (WA) and weighted sampling (WS) strategy using pre-assigned (P) and learned (L) weights respectively

In order to understand why the learned weights in the weighted sampling leads to significant improved performance compared to the pre-assigned weights, we compared the average tree performance and the ensemble (forest) performance for each of them. Figure 2 shows the error rates of average tree and ensemble of all the proposed weighting strategies in this study. It is obvious that using learned weights in the weighted sampling (LWS) leads to the strongest trees and consequently the best ensemble, given its lowest error rate. Moreover, if we compare the difference between the average tree performance and the ensemble performance for each of the weighting strategies, both strategies using learned weights yield bigger differences than using pre-assigned weights. It is interesting to note that, in the weighted aggregation strategy, the ensemble performance when using preassigned weights (PWS) is comparable to using learned weights (LWA), even if the average tree quality is higher in the former.

**Fig. 3:**
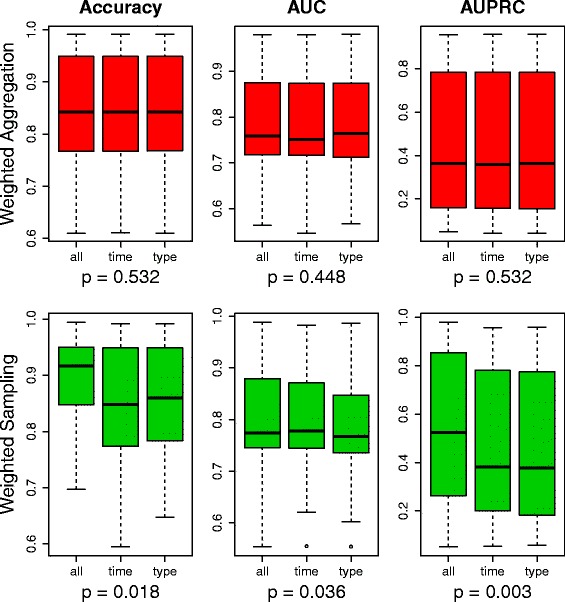
Compare weights learned on different specificity levels in terms of predictive performance. *Green* indicates a significant difference and *red* indicates no significance. *P*-values are obtained via a Friedman test

In the last experiment, we investigated the impact of the granularity of temporal weights learning, where three specificity levels were evaluated: (1) weights learned on a time-window level; (2) weights learned on an event-type level within each time window; and (3) weights learned on an individual level from each event type within each time window. Comparing the predictive performance of models exploiting weights from these three specificity levels in Fig. 3, we can see that the choice of specificity level has a significant impact on the predictive performance in the Weighted Sampling strategy, but no such significance is observed in the Weighted Aggregation strategy.

The post-hoc analysis through the Bergmann-Hommel procedure, as shown in Table 4, indicates that, overall, learning weights on each individual clinical event level (all) yields better performance compared to the other two more general levels; learning weights on each individual clinical event level (all) leads to significantly improved accuracy and AUPRC compared to learning weights per event type from each time window (type); the difference between learning weights per event type and per time window is almost negligible.

**Table 4:** Post-hoc analysis results of significance testing on pair-wised comparisons among different weights specificity levels in the weighted sampling strategy

	Accuracy				AUC				AUPRC		
ADE	all	type	time		all	type	time		all	type	time
all	–	0.052	0.045		–	0.028	0.746		–	0.002	0.144
type	–	–	0.626		–	–	0.028		–	–	0.052
Ave. Rank	1.53	2.16	2.32		1.68	2.53	1.79		1.47	2.58	1.95

## Discussion

This study evaluated the impact of learning temporal weights of clinical events, which was applied through two strategies, weighted aggregation and weighted sampling, in electronic health records for adverse drug event detection. The evaluation was done by exploiting these strategies to generate features on 19 datasets extracted from an EHR database. The results show that in the weighted sampling strategy, using learned weights significantly improves the predictive performance compared to using pre-assigned weights; however, there is no significant difference between them in the weighted aggregation strategy. One explanation for the latter could be the high sparsity of the data. All of the 19 datasets in this study are of high dimensionality and sparsity due to the fact that most clinical events only occurred to a small group of patients, i.e., the vast majority of the examples for a given feature have a value of zero. For features with only one or very few non-zero values, the impact of applying different weighting strategies is almost negligible, even though some of these features might be valuable indicators. To be more specific, if a feature has only one or a few non-zero values, when using it as an indicator to classify the examples, it does not matter whether these small numbers of non-zero values are weighted or not since they will most likely be distinguished against all the zero values; as a result, the applied weights will have almost no impact on the predictive performance. In the weighted aggregation strategy, the learned weights were embedded in the feature values, which does not affect the sparsity; therefore, for very sparse features, no matter how their weights change, their contribution to the predictive performance will not be influenced. However, in the weighted sampling strategy, the learned weights were used as sampling probabilities, which makes use of the higher weighted, i.e., more informative, features more often, so that the weights have a higher and more direct impact on the predictive performance.

Besides the difference between the learned weights and the pre-assigned weights within each strategy, the weighted sampling strategy only leads to better predictive performance than the weighted aggregation strategy when using learned weights, i.e., the choice of strategy on applying the weights matters less with the pre-assigned weights (given the close results between PWA and PWS). This indicates that the improved predictive performance is a result of the combination of learning weights and application of the weighted sampling strategy. To understand more deeply how the performance is improved, we looked into the random forest models in terms of average tree quality and diversity. The results in Fig. 2 indicates that using learned weights in the weighted sampling strategy yields the best predictive performance due both to better individual tree quality and larger diversity. Moreover, learning weights also contributes to bigger diversity in the weighted aggregation strategy. Comparing learning weights in both the weighted aggregation (LWA) and weighted sampling (LWS) strategy, it seems that the latter does not result in a lower diversity as one would have expected, given that, there, the important features appear in many trees. It is probably due to the high dimensionality of all the datasets and, more importantly, an indication that a really small portion of features are relevant/important; hence, even though this small number of important features appear in many trees, the diversity is still maintained by the other features that are selected to build each tree. Therefore, weighted sampling using learned weights is particularly beneficial when using vast amounts of clinical events from EHRs for predictive modelling, where probably only a small amount of information is highly relevant to the predicting target, while the remaining information is less useful.

Another advantage of learning weights, besides taking into account the importance of each clinical event, is that weights can be learned on a more specific level: rather than just looking at the time window, the specificity can be adjusted. Concerning the granularity of weights, pre-assigning weights is equivalent to learning weights per time window (denoted as *time* in Fig. 3 and Table 4), both of which assign the same weight to all clinical events from the same time window. Here, within a time window, different weights can be learned further for each event type (denoted as *type*) – i.e., diagnosis, drug and clinical measurement – or even each individual clinical event within each event type (denoted as *all*). Again, the impact of using weights from different specificity levels yields a significant impact only in the weighted sampling strategy, but not in the weighted aggregation strategy. It is probably a result of the high dimensionality and sparsity of the data as argued above. By looking at the post-hoc analysis results for the pairwise comparisons, we can see that, on one hand, learning weights for each event type gives the worst performance, which suggests that there is no evidence for treating different event types differently; on the other hand, learning weights for each individual clinical event, the most specific level, yields the best performance, which indicates that individual events should not be treated in the same way. For instance, diagnoses in general shouldn’t be more or less important than drugs; however, a particular diagnosis code can be more important than a particular drug. It is interesting to note that it is not that more specific weights learning gives better performance, as one would expect. Weights on the *type* level are more specific than the ones on the *time* level, but the latter sometimes outperforms the former.

One limitation of this study is that only one learning weights algorithm, namely random forest’s variable importance, is explored. In future work, other methods are worth investigating; for example, methods that do not use feedback from a classifier to assign weights but use a pre-existing model instead, such as information gain. Further along this line, learning weights here focuses on temporality and informativeness of the clinical events; nevertheless, there are also other criteria that can be used to learn the weights. For example, clinical events are represented as the number of times that they occur in a time window, which disallows learning weights based on event values and/or how sharply an event value changes over time. This is especially crucial when using clinical measurements as features.

## Conclusion

The temporal information that is embedded in longitudinal healthcare data is very valuable for building predictive models for pharmacovigilance. Electronic health records is one of this kind, where clinical events, such as diagnoses, drug administrations and clinical measurements, are typically time stamped. Using pre-assigned weights according to events’ time stamps has been shown to be an effective way of exploiting temporality in predictive models. However, pre-assigned weights are limited to the chronology of clinical events, but fails to take into account their importance, in terms of informativeness, when building the predictive models. To mitigate this problem, this study focuses on how to learn the weights automatically when constructing the predictive models in a way that reflects both temporality and importance of the clinical events. To take into account the temporality, each patient’s medical history is segmented into a set of time windows and features are extracted from each of them; for the importance, a random forest model is built using these extracted features to produce variable importance, which is then used as the weight for each feature. To apply these weights, two strategies are proposed: weighted aggregation and weighted sampling. The former aggregates the weighted clinical events from different time windows to form new features, while the latter keeps the original features but samples them with their weights as probabilities when building each tree in the forest. We conclude here that learning weights is significantly beneficial in terms of predictive performance in the weighted sampling strategy. Moreover, weighted aggregation generally diminishes the impact of temporal weighting of the clinical events, irrespective of whether the weights are pre-assigned or learned.
